# 3D-Printed Cellulose Aerogels Minimally Cross-Linked with Polyurea: A Robust Strategy for Tissue Engineering

**DOI:** 10.1021/acsami.5c08389

**Published:** 2025-05-28

**Authors:** Ana Iglesias-Mejuto, Grigorios Raptopoulos, Nanthilde Malandain, Mariana Neves Amaral, Inés Ardao, Matjaž Finšgar, Anna Laromaine, Anna Roig, Catarina Pinto Reis, Carlos A. García-González, Patrina Paraskevopoulou

**Affiliations:** a AerogelsLab, I+D Farma Group (GI-1645), Department of Pharmacology, Pharmacy and Pharmaceutical Technology, Faculty of Pharmacy, iMATUS and Health Research Institute of Santiago de Compostela (IDIS), 16780 Universidade de Santiago de Compostela, Santiago de Compostela E-15782, Spain; b Institut de Ciència de Materials de Barcelona, ICMAB-CSIC, Campus UAB, Bellaterra 08193, Spain; c Inorganic Chemistry Laboratory, Department of Chemistry, 68993 National and Kapodistrian University of Athens, Panepistimiopolis Zografou, Athens 15771, Greece; e Research Institute for Medicines (iMed. ULisboa), Faculty of Pharmacy, Universidade de Lisboa, Av. Professor Gama Pinto, Lisboa 1649-003, Portugal; f Instituto de Biofísica e Engenharia Biomédica (IBEB), Faculdade de Ciências, Universidade de Lisboa, Campo Grande, Lisboa 1749-016, Portugal; g BioFarma Research Group, Department of Pharmacology, Pharmacy and Pharmaceutical Technology, Innopharma Drug Screening and Pharmacogenomics Platform, Centro Singular de Investigación en Medicina Molecular y Enfermedades Crónicas (CiMUS), 16780 Universidade de Santiago de Compostela, Santiago de Compostela 15782, Spain; h Faculty of Chemistry and Chemical Engineering, 54765 University of Maribor, Smetanova ulica 17, Maribor SI-2000, Slovenia

**Keywords:** 3D printing, aerogels, cellulose, methylcellulose, polyurea, tissue engineering, X-aerogels

## Abstract

Cellulose and its derivatives are increasingly explored in biomedical applications due to their biocompatibility, biodegradability, and mechanical performance. In regenerative medicine, aerogel scaffolds with tunable morphology and composition are highly valued for their ability to support tissue regeneration. Three-dimensional (3D) printing offers an effective method to fabricate aerogels with hierarchical pore structures, comprising interconnected macropores and mesopores, that are crucial for tissue engineering. For clinical use, 3D printing should ensure the structural integrity of printed structures and achieve a printing resolution that allows for customization. In this work, the X-aerogel technology, implemented via polyurea cross-linking, was applied to 3D-printed cellulose structures, thereby expanding the potential applications of both technologies. Specifically, 3D-printed methylcellulose (MC) and MC doped with bacterial cellulose nanofiber (MCBCf) gels were cross-linked with an aliphatic polyurea, yielding, after supercritical drying, the corresponding (X-MC and X-MCBCf) aerogels. Elaborate characterization with ATR-FTIR, XPS, ToF-SIMS, N_2_ porosimetry, He pycnometry, and SEM confirmed the formation of polyurea on the biopolymer framework, reinforcing the structure and improving the mechanical properties without altering the morphology or textural characteristics of the materials. A significant outcome of cross-linking with polyurea is the long-term stability of X-MC and X-MCBCf aerogels in water, in contrast to their native counterparts, and their capacity to absorb water up to 1800% w/w within only 2 h. Preliminary biological evaluation of the materials, including *in vitro* (cell compatibility, hemolytic activity), *in ovo* (HET-CAM), and *in vivo* (A. salina model) tests, showed good cell viability, blood compatibility, and safety for living organisms. From a fundamental materials perspective, the most important finding of this work is the disproportionally high stability of X-MC and X-MCBCf in physiological environments, achieved with only a minimal (almost undetectable) amount of cross-linking polyurea. From an application standpoint, the findings of this study, collectively, position these aerogels as sustainable and promising candidates for tissue engineering scaffolds.

## Graphical abstract

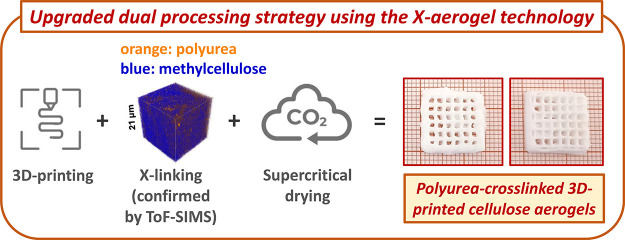

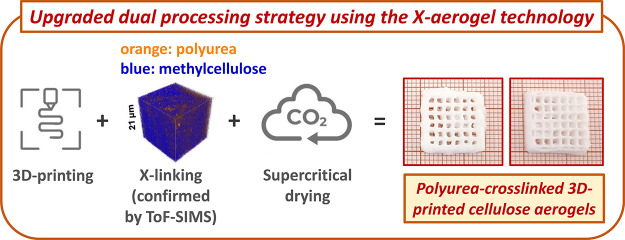

## 1 Introduction

Tissue engineering is an emerging field that aims to replace damaged tissues using scaffolds, living cells and bioactive molecules to reestablish the native tissular functionality^1^ and reduce autografted tissue shortages.^2^ The design and development of highly porous biomaterials with mesopores and macropores interconnected within three-dimensional (3D) nanostructures is a field that holds great potential in regenerative medicine to efficiently promote cell growth and nutrient diffusion simultaneously.^3,4^ A dual processing strategy, combining 3D-printing and supercritical fluid drying, has been recently developed to manufacture aerogels with bimodal porosity and suitable functionality for tissue healing.^5^

Aerogels are lightweight nanostructured nanoporous materials with open porosity and high specific surface area, which can be obtained after the controlled removal of the pore-filling fluid from a gel without significant structural change or volume reduction.^6–8^ Aerogels can be inorganic, organic or hybrids thereof.^9^ Among them, biopolymer aerogels are increasingly attracting attention due to their origin from natural resources, environmentally friendly preparation methods, and diverse applications across various fields, including environmental remediation and biomedicine.^10^ In the context of biomedicine, biopolymer aerogel scaffolds can be employed as scaffolds for tissue repair, as their architectures can be tailored to mimic the natural extracellular matrix (ECM).^11^ In addition to biomimicry, certain aerogel properties, such as high porosity, low swelling or shrinkage, or their efficiency in dry interfaces, like the ones aimed in wounds, can improve cell attachment and nutrient transportation. Furthermore, their outstanding fluid uptake capacity enhances their effectiveness at wound interfaces.^11,12^

Cellulose is a biocompatible, biodegradable, sustainable and environmentally friendly biopolymer, and the most prevalent on Earth.^13^ Cellulose aerogels have a wide range of applications across several sectors,^3^ including energy, food and textile industries, as well as biomedicine, with drug delivery and both hard and soft tissue engineering being the most promising fields.^14,15^ They are typically endowed with mesopores and small macropores;^16^ techniques like 3D-printing can provide the large macropores commonly required for tissue engineering, thus inducing a positive long-term biological response.^17^ The characteristics of cellulose can be enhanced either by engaging cross-linking strategies, or by chemical (functional group) modification yielding numerous cellulose derivatives such as methylcellulose (MC).^18,19^ Cellulose from bacterial origin, or bacterial cellulose (BC), also presents potential in tissue engineering for nerve, bone, cartilage, or vascular repair,^1,15,20^ due to its nanoporous and fibrillar structure that resembles native ECM, as well as its purity, water-holding capacity and mechanical performance.^1,21^ BC can be combined with different cellulose derivatives to obtain BC composites with potential as biomimetic scaffolds for tissue healing.^3,22,23^ Also, by the technological integration of 3D-printing and supercritical fluid drying, BC-containing aerogels with mesopores and large macropores interconnected in the same nanostructure can be obtained.^24^

Current challenges for the clinical application of 3D-printed scaffolds include achieving sufficient structural integrity and printing resolution to enable customization.^2^ Different cross-linking methods have been proposed to promote the reinforcement of 3D-printed scaffolds; for example, the use of glutaraldehyde vapor as a postprocessing step in the manufacturing process of calcium alginate/hydroxyapatite aerogels.^25^ The different layers of the glutaraldehyde-cross-linked aerogels have been perfectly arranged in the 3D-printed structure, but with a slight negative effect on certain physicochemical properties, i.e., their specific surface area and porosity. Among cross-linking strategies, cross-linking with polyurea at the nanoscopic rather than the molecular level (referred to as the X-aerogel technology),^26–28^ emerges as an extremely robust option for the reinforcement of the porous structure of wet gels and aerogels.^29^

Polyurea is a stable polymer and the corresponding aerogels have been obtained in several form factors and morphologies via polymerization of multifunctional isocyanates with multifunctional amines,^30,31^ water (via *in situ* formation of amines),^32,33^ or mineral acids.^34^ The X-aerogel technology was introduced in the early 2000s as an efficient method to enhance the mechanical properties of silica^26–28^ and other inorganic (e.g., oxides, ceramics)^35–41^ aerogels, as well as of certain synthetic polymers.^42–44^ In 2020, the X-aerogel technology, implemented with polyurea as the cross-linking polymer, was expanded to biopolymer aerogels (alginates and chitosan).^45–47^ In general, polyurea-cross-linked aerogels can be obtained from preformed inorganic or biopolymer gels via reaction of the surface functional groups with triisocyanates and their subsequent polymerization with water adsorbed on the surface of the gel network, leading to the formation of a polymeric nanostructure over the inorganic or biopolymer skeleton. X-aerogels present low shrinkage, high porosity, high specific surface areas and enhanced mechanical properties, as well as the intrinsic-by-definition open and nanoporous structure of aerogels and all the good characteristics of the biopolymers (e.g., biocompatibility). Therefore, they are very promising materials for environmental^48–51^ and biomedical^52,53^ applications. Given the desirable combination of material properties, along with the versatility and the expanding applications for polyurea-based materials,^30,54^ it has been shown that X-alginate aerogels have great potential for fundamental research and practical applications.

The combination of X-aerogel technology with 3D-printing has the potential to furnish personalized scaffolds with enhanced structural integrity and printing fidelity. In this context, choosing cellulose derivatives, such as MC and BC nanofibers (BCf) as the aerogel skeletal backbone can lead to reinforced porous structures with interconnected mesopores and macropores that mimic the native ECM and thus represent improved natural alternatives for soft and hard tissue engineering. Besides, both technologies are quite new in the preparation of biopolymer-based aerogels, with only a few prior examples in the literature, as outlined above, and have never been used together before.

In this work, cross-linking with polyurea was applied to 3D-printed cellulose structures, thus extending the application of X-aerogel technology to personalized biopolymer aerogel scaffolds. For this purpose, 3D-printed MC and MCBCf gels were reacted with an aliphatic triisocyanate and were cross-linked with the corresponding aliphatic polyurea. Afterward, supercritical drying was applied to yield polyurea-cross-linked 3D-printed cellulose (X-MC and X-MCBCf) aerogels. The characterization of these aerogels was performed using Attenuated Total Reflectance Fourier Transform Infrared Spectroscopy (ATR-FTIR), X-ray Photoelectron Spectroscopy (XPS), Time-of-Flight Secondary Ion Mass Spectrometry (ToF-SIMS), N_2_ porosimetry, He pycnometry, Scanning Electron Microscopy (SEM) and Transmission Electron Microscopy (TEM). Water contact angle and water uptake measurements were performed to assess the wetting properties of the materials. X-MC and X-MCBCf aerogels were also evaluated through several *in vitro* (cell compatibility, hemolytic activity), *in ovo* (Hen’s Egg Test on the Chorioallantoic Membrane (HET-CAM)) and preliminary *in vivo* (*Artemia salina* model) tests to ensure their standards and safety to sustain *in vivo* performance. From a fundamental materials perspective, the most significant finding of this work is the disproportionally high stability of X-aerogels in physiological environments, achieved with only a minimal (barely detectable) amount of cross-linking polymer (i.e., polyurea). From the applications perspective, the aerogels presented here offer a sustainable and promising option for tissue engineering scaffolds.

## 2 Experimental Section

### 2.1 Materials

Methylcellulose (MC, viscosity 15 cps, *M*_w_ 14 kDa, DS 1.5–1.9), citric acid monohydrate, sodium hydroxide (NaOH), benzyl alcohol, iron­(III) acetylacetonate (Fe­(acac)_3_, 97%), and resazurin (7-hydroxy-3H-phenoxazin-3-one-10-oxide) were purchased from Sigma-Aldrich (Steinheim, Germany). Absolute ethanol (EtOH) was provided by VWR (Radnor, PA, USA). Water was purified by reverse osmosis (resistivity >18 MΩ·cm; Milli-Q, Millipore, Madrid, Spain). The aliphatic triisocyanate Desmodur Ultra N3300 was kindly provided by Covestro AG (Leverkusen, Germany). Acetonitrile (MeCN, HPLC grade) and acetone were provided by Fisher (Madrid, Spain). All solvents were used as received.

*Komagataeibacter xylinus* bacterial strain (NCIMB 5346) was supplied by the Spanish Type Culture Collection (CECT, Spain). Hestrin-Schramm (HS) culture medium was prepared with sodium phosphate dodecahydrate, citric acid monohydrate, dextrose, peptone and yeast extract provided by Condalab (Madrid, Spain). *Artemia salina* eggs and seawater salt were obtained from JBL GmbH and Co., KG (Neuhofen, Germany).

### 2.2 Methods

#### 2.2.1 Preparation of Bacterial Cellulose Nanofibers (BCf)

BCf were prepared following well-established protocols.^21,55^ In brief, *K. xylinus* was grown in HS medium (5 mL) and incubated statically (7 days, 30 °C). Bacterial broth (4 mL) was mixed with HS medium (56 mL). After incubation (5 days, 30 °C) BC pellicles were cleaned with EtOH (50% v/v, 10 min), boiled twice in Milli-Q water (20 min) and twice in 0.1 M NaOH (20 min, 90 °C). The obtained BC films were autoclaved (30 min, 121 °C) and blended in Milli-Q water (1 L) at the maximum speed of a commercial blender (Jata electro Mod. BT1200, Tudela, Spain). BCf were then filtered with a Stericup Quick Release Filter Millipore with a PES membrane (0.22 μm), autoclaved (30 min, 121 °C) and dispersed in Milli-Q water (12 mg mL^–1^).

#### 2.2.2 Preparation of Bacterial Cellulose Nanofibers (BCf) Doped with Superparamagnetic Iron Oxide Nanoparticles (SPIONs)

BCf (15 g) were mixed overnight with benzyl alcohol (40 mL) and Fe­(acac)_3_ (1.1 g). The mixture was then heated for 5 min at 60 °C and for 10 min at 210 °C in a microwave oven (Milestone, Sorisole, Italy) at a frequency of 2.45 GHz, a power of 750 W and 5% agitation. The mixture was cooled down and BCf doped with superparamagnetic iron oxide nanoparticles (SPIONs) were filtered, washed twice with acetone and once with Milli-Q water, and autoclaved (121 °C for 20 min).

#### 2.2.3 Process Manufacturing of Polyurea-Cross-Linked 3D-Printed Cellulose Aerogels

MC and MCBCf alcogels and aerogels were prepared following well-established protocols.^24^ MC aqueous inks (12% w/w) were obtained by using a homogenizer (VWR vos 60, Radnor, PA, USA) at 600 rpm for 1 h at room temperature and were degassed for 10 min in a sonication bath (Branson 3510 Emerson, Ferguson, MO, USA).

Alternatively, a second ink formulation incorporating a BCf suspension (6 mg mL^–1^, 20% v/v) during the first step of the ink preparation was employed. Specifically, BCf were mixed with the MC ink by using a homogenizer (VWR vos 60, Radnor, PA, USA) at 600 rpm for 1 h at room temperature. The ink was degassed as described above. Inks containing SPIONs-doped BCf were prepared with the same concentration and following the same procedure.

3D-printed hydrogels were obtained from these inks using a Cellink BIOX Bioprinter (Boston, MA, USA) with an extrusion printhead and a 3 mL syringe with a 410-μm nozzle. Scaffolds with a grid pattern of 20 × 20 × 3 mm and 8 layers were printed at 3 mm s^–1^, 40 °C and 40 kPa and immersed in ethanol (MC and MCBCf alcogels).

Subsequently, cross-linking with polyurea was carried out following well-established protocols.^45^ MC and MCBCf alcogels were solvent exchanged with dry MeCN (4 times) before being immersed in a solution of the triisocyanate (Desmodur N3300 Ultra, 0.081 M) in dry MeCN (4 times the volume of the gels). Samples were kept in that solution at room temperature for 24 h and at 70 °C for 72 h. Polyurea-cross-linked gels were finally solvent exchanged with dry acetone (4 times), and then placed into an autoclave (Model E3100, Quorum Technologies, East Sussex, UK) to perform drying with supercritical CO_2_. The autoclave was filled with CO_2_ and acetone was drained out when displaced by CO_2_ (5 times, 30 min each). The autoclave was set at 45 °C and it was maintained for 1 h before CO_2_ was slowly released. The corresponding polyurea-cross-linked 3D-printed cellulose aerogels are referred to as *X-MC* and *X-MCBCf* aerogels.

Control aerogel samples, referred to as *MC*, *MCBCf* and *MCBCf-SPIONs* aerogels, were prepared from the corresponding alcogels after solvent exchange with dry acetone (4 times) and drying with supercritical CO_2_ (as described above).

#### 2.2.4 Physicochemical Characterization

Bulk densities (ρ_*b*_) of the aerogels were obtained from their weight and physical dimensions, according to eq 1. Skeletal densities (ρ_*s*_) were measured using He pycnometry with a Micromeritics AccuPyc II 1340 pycnometer (Micromeritics, Norcross, GA, USA). Overall porosity and the total pore volume were calculated following eqs 2 and 3, respectively. The printing fidelity of the aerogels was assessed by calculating the shape fidelity factor (SFF), according to eq 4, where CAD is the computer aided design area, as defined in the printing file.

$$\rho_{b}=\mathrm{aerogel}\;\mathrm{mass}/\mathrm{aerogel}\;\mathrm{volume}$$ 1

$$\Pi =\left(\rho_{s}-\rho_{b}\right)/\rho_{s}$$ 2

$$V_{\mathrm{Total}}=1/\rho_{b}-1/\rho_{s}$$ 3

SFF=alcogeloraerogelprintedarea/CADarea 4

N_2_ porosimetry was performed on a Micromeritics Tristar II 3020 surface area and porosity analyzer (Micromeritics, Norcross, GA, USA). All samples were degassed at 80 °C for 24 h using Micromeritics VacPrep 061 (Micromeritics, Norcross, GA, USA).

SEM characterization was performed using an EVO LS15 instrument (Zeiss, Oberkochen, Germany). Samples were sputtered with iridium. Confocal microscopy was performed with a Leica TCS-SP2 spectral confocal microscope (Leica TCS-SP2, Leica Microsystems Heidelberg GmbH, Mannheim, Germany) at 405 nm. TEM characterization was performed using a JEOL JEM-2010 instrument (JEOL, Tokyo, Japan) operating at 200 kV.

ATR-FTIR spectra were obtained with a Shimadzu FTIR IRAffinity-1 spectrometer equipped with an ATR Shimadzu QATR10 single-reflection attachment. Spectra were recorded in the wavenumber range 400–4000 cm^–1^.

XPS measurements were conducted using a Supra+ device (Kratos, Manchester, UK) equipped with an Al K_α_ X-ray source. A charge neutralizer was activated throughout the measurements. Spectra calibration was performed by referencing the C–C/C–H peak in the C 1s spectrum at 284.8 eV. XPS spectra were acquired at a 90° takeoff angle with a 300 by 700 μm analysis area. A pass energy of 20 eV was used for high-resolution spectra, while 160 eV was applied for survey spectra. Data acquisition and processing were carried out using ESCApe 1.5 software (Kratos). Samples were mounted on an XPS holder with double-sided carbon tape.

ToF-SIMS measurements were conducted using an M6 device. A 30 keV Bi_3_^+^ primary ion beam was applied at a target current of 0.6 pA. For sputtering, an Ar_1000_^+^ gas cluster ion beam (GCIB) was utilized with an acceleration energy of 10 keV and a target current of 10 nA. To mitigate surface charging during analysis, a flood gun was activated, and the main analysis chamber was flooded with Ar gas at a final pressure of 5·10^–7^ Torr. A surface potential was applied as necessary. Data acquisition and processing were performed using SurfaceLab 7.3 software (IONTOF, Münster, Germany). GCIB sputtering was conducted over a 500 by 500 μm area, while analyses were carried out in the center of the sputtered region over a 200 by 200 μm area. Spectra were calibrated using peaks at known mass-to-charge (*m*/*z*) ratio, i.e. C^–^ at *m*/*z* 12.00, C_2_^–^ at *m*/*z* 24.00, C_3_^–^ at *m*/*z* 36.00, and C_4_^–^ at *m*/*z* 48.00. To determine the depth of the sputter crater, DektakXT stylus profilometer was used (Bruker, Karlsruhe, Germany).

Water contact angle measurements were performed by placing a 9 μL droplet of Milli-Q water on the surface of the aerogels while recording the droplet shape. The water contact angle was calculated using ImageJ software (2 measurements per replicate, 4 replicates per formulation). The water uptake was calculated by placing aerogel samples (0.5 × 0.5 × 0.3 cm) in Eppendorf tubes containing 5 mL of Milli-Q water, at 37 °C and 50 rpm for 10 days. At selected time intervals, samples were taken out, weighed using an analytical balance, and reimmersed in water in the Eppendorf tubes. Any excess water on their surface was carefully wiped out before the measurement. The water uptake was calculated according to eq 5. The reported values are the average of at least three measurements.

WaterUptake(%)=(massofwetsample−massofaerogel)/massofaerogel×100 5

#### 2.2.5 Biological Evaluation

The biological evaluation of polyurea-cross-linked 3D-printed cellulose aerogels was carried out following well-stablished protocols for the characterization of aerogels regarding their cytocompatibility, hemolytic activity, HET-CAM, and preliminary safety tests.^5,19,24^

##### Cytocompatibility Tests

The cytocompatibility of X-MC and X-MCBCf aerogels was evaluated by measuring the viability of mouse embryo fibroblasts (NIH/3T3) after contact with aerogel samples. Cells were seeded at a concentration of 12,000 cells cm^–2^ in 24-well plates with Dulbecco’s Modified Eagle’s Medium (600 μL) supplemented with bovine calf serum (10% v/v), streptomycin (100 g mL^–1^) and penicillin (100 U mL^–1^). Aerogel samples (0.5 × 0.5 × 0.3 cm) were then UV-sterilized (30 min) and placed in culture inserts in contact with the cells. After incubation at 37 °C in a humidified atmosphere enriched with CO_2_ (5% v/v) for 24 or 72 h, the samples were removed, the growth medium was aspirated and a resazurin solution in supplemented growth medium (44 μM, 100 μL) was added into each well. After incubation under the same experimental conditions for 3 h, fluorescence was measured (excitation λ = 544 nm; emission λ = 590 nm) in a microplate reader (Infinite M200, Tecan Group Ltd., Männedorf, Switzerland). The resazurin solution was used as a blank and cells incubated at the same experimental conditions without contact with aerogels were used as positive controls. All tests were run in triplicate. The viability of the cells was calculated according to eq 6, where Abs_s_ is the absorbance of the supernatant with X-MC and X-MCBCf aerogels and Abs_p_ is the absorbance of the positive control (100% cell viability). The reported values are the average of at least three measurements.

$$\mathrm{Cell}\;\mathrm{viability}(\%)={\mathrm{Abs}}_{s}/{\mathrm{Abs}}_{p}\times 100$$ 6

##### Hemolytic Activity Tests

Human blood (Galician Transfusion Center, Santiago de Compostela, Spain) was obtained following the principles of the Declaration of Helsinki. Fresh blood was diluted in a NaCl solution (0.9% w/v) to a concentration of 3% v/v. The diluted blood (1 mL) was poured into Eppendorf tubes containing phosphate buffer saline (PBS, pH 7.4, 100 μL; negative control), Triton X-100 (4% v/v, 100 μL; positive control) or X-MC and X-MCBCf aerogel samples (0.5 × 0.5 × 0.3 cm). The tubes were incubated (37 °C, 60 min, 100 rpm) and centrifuged (10.000 g, 10 min; Sigma 2–16P, Sigma Laboratory Centrifuges, Osterode am Harz, Germany). The absorbance of the hemoglobin was measured in the supernatant at λ = 540 nm (FLUOStar Optima, BMG Labtech, Ortenberg, Germany). All tests were run in triplicate and hemolysis was calculated according to eq 7, where Abs_s_ is the absorbance of the supernatant with X-MC and X-MCBCf aerogels, Abs_n_ is the absorbance of the negative control (0% hemolysis) and Abs_p_ is the absorbance of the positive control (100% hemolysis).

$$\mathrm{Hemolysis}\left(\%\right)=\left({\mathrm{Abs}}_{s}-{\mathrm{Abs}}_{n}\right)/\left({\mathrm{Abs}}_{p}-{\mathrm{Abs}}_{n}\right)\times 100$$ 7

##### Hen’s Egg Test on the Chorioallantoic Membrane (HET-CAM) Test

HET-CAM evaluated X-MC and X-MCBCf aerogels following the Interagency Coordinating Committee on the Validation of Alternative Methods (ICCVAM) guidelines. Fertilized hens’ eggs (50–60 g, Coren, Ourense, Spain) were incubated (37 °C, 60% humidity) with an 8 h scheduled rotation incubator (Ineltec CC SR 0150, Barcelona, Spain). A small window was opened on day 9 to access CAM and add X-MC and X-MCBCf aerogel samples (0.5 × 0.5 × 0.3 cm), PBS (pH 7.4, 300 μL, negative control) or NaOH (0.1 N, 300 μL, positive control). All tests were run in triplicate. CAM vessels were observed for 5 min after contact with the aerogel samples to check the appearance of hemorrhage, vascular lysis, or clotting (visual inspection).

##### Preliminary Safety Test

Commercial seawater salt was dissolved in tap water following the supplier’s recommendations for preparing artificial seawater. *A. salina* eggs were left therein to hatch for 48 h under aeration and illumination at 25–30 °C. Artificial seawater (900 μL) with 10–15 nauplii was poured into each well of a 24-well plate. X-MC and X-MCBCf aerogel samples (0.5 × 0.5 × 0.3 cm), artificial seawater (100 μL; negative control) or dimethyl sulfoxide (DMSO, 100 μL; positive control) were added, and were incubated for 24 h under the same experimental conditions. The dead nauplii were counted. Afterward, DMSO (100 μL) was pipetted to kill all living nauplii. All tests were run using four replicates and mortality was calculated according to eq 8, where “Dead 24 h” is the number of dead nauplii after 24 h in contact with the aerogel samples and “Dead Total” is the total number of nauplii per well.

Mortality(%)=(Dead24h)/(DeadTotal)×100 8

##### Statistical Analysis

All results were reported as mean value ± standard deviation, and post hoc Tukey HSD multiple comparison tests were carried out to assess the statistical significance of differences among the different experiments. Values of *p* < 0.05 were considered statistically significant.

## 3 Results and Discussion

### 3.1 Preparation and Visual Characterization of Polyurea-Cross-Linked 3D-Printed Cellulose Aerogels

The dual processing strategy combining 3D-printing of gels and their subsequent supercritical fluid drying^56^ to yield 3D-printed aerogels has been upgraded with the polyurea-cross-linking technology (X-aerogel technology).^45–47^ More specifically, 3D-printed cellulose gels were prepared using inks of methylcellulose (MC; Figure 1a) or methylcellulose with dispersed bacterial cellulose nanofibers (MCBCf; Figure 1a) and printing in ethanol (MC and MCBCf alcogels).^24^ 3D-printing is an effective method to fabricate aerogels with hierarchical pore structures comprising interconnected macropores and mesopores, both crucial for tissue engineering.^24,56^ BCf were added into 3D-printed aerogels in order to increase the printing fidelity and to decrease the volume shrinkage of the aerogels. The concentration of BCf used in the inks for 3D-printing corresponds to the best-performing formulation identified in a previous study.^24^ Lower concentrations than those employed in this work resulted in 3D-printed aerogels with poor printing fidelity. Moreover, it was demonstrated that this selected BCf content did not negatively impact the physicochemical or biological performance of the resulting cellulose-in-cellulose aerogels. All gels were subsequently solvent exchanged with dry MeCN and kept in a solution of the aliphatic triisocyanate (Desmodur N3300, 0.081 M; Figure 1a) in dry MeCN at room temperature for 24 h and then at 70 °C for 72 h to complete the cross-linking process (i.e., the formation of polyurea). The proposed mechanism, based on previous studies with biopolymers bearing the same functional groups (i.e., – OH)^45^ and the characterization data discussed in Section 3.2, is summarized in Figure 1b. Briefly, the triisocyanate reacts with – OH groups on the surface of the cellulose skeleton and gets attached to it via the formation of urethane groups. The unreacted isocyanate groups react with water adsorbed on the cellulose skeleton, yielding amines, which react fast with fresh triisocyanate that is present in the pores to form polyurea. Polyurea-cross-linked gels were finally solvent exchanged with dry acetone and dried with supercritical CO_2_ to yield the corresponding polyurea-cross-linked 3D-printed cellulose aerogels (referred to as X-MC and X-MCBCf aerogels; Figure 1c). Native MC and MCBCf aerogels (Figure 2a) were also prepared, for comparison purposes, from MC and MCBCf alcogels, after solvent-exchange with dry acetone and supercritical drying.

**Figure 1:**
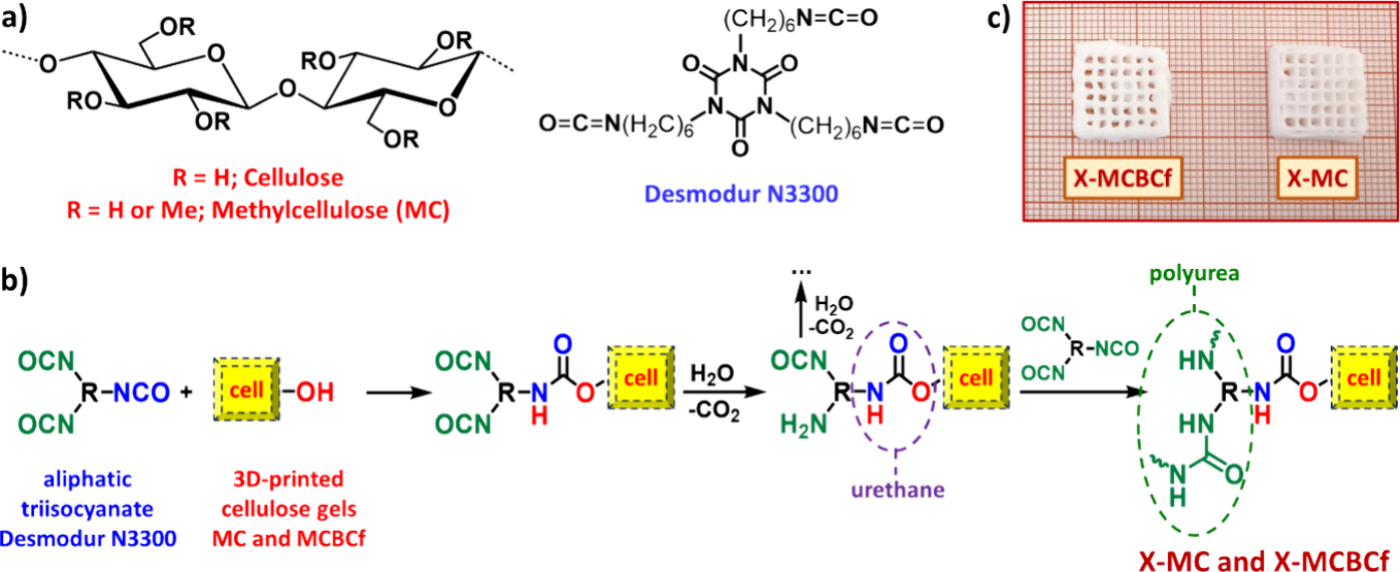
(a) Chemical structures of cellulose, methylcellulose (MC) and Desmodur N3300. (b) Mechanism of cross-linking of preformed cellulose (MC and MCBCf) gels with polyurea. (c) Optical photograph of polyurea-cross-linked (X-MC and X-MCBCf) 3D-printed cellulose aerogels.

**Figure 2:**
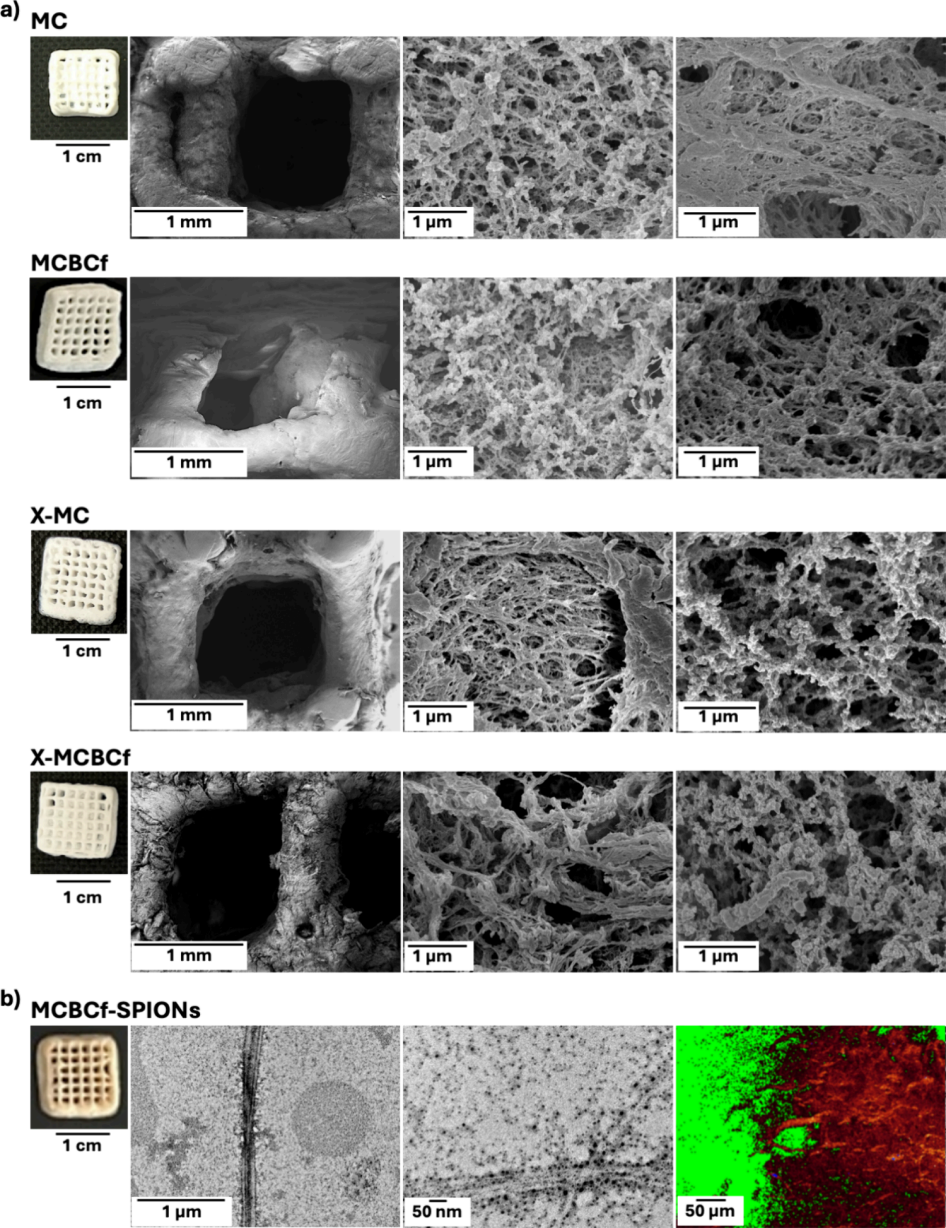
(a) Optical photographs and SEM images of native (MC and MCBCf) and polyurea-cross-linked (X-MC and X-MCBCf) 3D-printed aerogels imaged at three different magnifications (500, 50.000 and 75.000×). (b) Optical photograph, TEM at two different magnifications (5.000 and 20.000×, respectively) and confocal microscopy images observed at 405 nm of SPIONs-doped MCBCf (MCBCf-SPIONs) aerogels.

Nano-cross-linking with polyurea did not affect the 3D-printed structure of cellulose aerogels, as shown in the optical photographs of Figures 1c and 2a and in SEM images (Figure 2a), which show that the filaments are perfectly aligned in the scaffolds. Mesopores and macropores were homogeneously allocated through the nanoporous structure of all aerogels (MC, MCBCf, X-MC and X-MCBCf); the distribution of the different pore sizes was also maintained after cross-linking (Figure 2a), thereby suggesting that this functionalization method is a good strategy to promote structural integrity without an adverse effect on the morphological features of the 3D-printed scaffolds and certainly showing advantages over dense filaments for tissue regeneration.^57^ This finding is in agreement with previous observations on polyurea-cross-linked biopolymer (i.e., alginates and chitosan) aerogels,^45,47^ in which cases the morphology of these aerogels was for all practical purposes identical with that of the corresponding native aerogels, since polyurea forms a nanothin layer over the entire skeleton of the biopolymer. In particular, aliphatic polyurea from Desmodur N3300 has been found (from SANS data) to uniformly coat the primary particles of the biopolymer following the contours of the biopolymer skeleton.^58^

BCf also preserved their integrity and random distribution in the aerogel matrix (Figure 2b). It has been reported that certain cellulose derivatives can induce ink instability, due to inhomogeneous dispersion in the ink matrix (such as cellulose nanocrystals in polylactic acid matrices) during the 3D-printing process, which decreases the quality and resolution of the 3D-printed structures.^59^ This problem has been overcome in this work through the efficient homogenization of BCf in the 3D-printing inks. To demonstrate that BCf have been homogeneously distributed in the material, SPIONs-doped BCf were incorporated in the ink and used to prepare MCBCf aerogels doped with SPIONS (Figure 2b). Indeed, a homogeneous orange-color was detected in the MCBCf-SPIONs scaffolds, thereby demonstrating the presence of SPIONs-doped BCf in the 3D-printed aerogels (Figure 2b). Confocal microscopy analysis showed homogeneous fluorescence throughout the analyzed sample, and no black regions due to concentration of SPIONs,^55^ supporting homogeneous distribution of SPIONs and therefore BCf in the aerogel.

### 3.2 Chemical Characterization

An extensive chemical characterization of polyurea-cross-linked 3D-printed aerogels was performed to confirm the formation of polyurea and the effect of this processing step on the physicochemical properties of the scaffolds. ATR-FTIR spectra of X-MC and X- MCBCf aerogels (Figure 3) exhibited the stretching vibration of the carbonyl groups of the isocyanurate ring (1686 cm^–1^) as well as the scissoring vibration linked to the N–H groups (1541 cm^–1^), in agreement with the literature.^31,45^ Other characteristic vibrations of polyurea (e.g., the stretching vibration of the carbonyl groups of the urea groups at 1636 cm^–1^) fall within the vibrations of MC, and therefore, they cannot be distinguished. In addition, the absence of bands at 2266 cm^–1^ (that would be assigned to the –NCO group) showed that no unreacted triisocyanate has remained in the aerogels. These findings indicate that polyurea was indeed uptaken by the cellulose skeleton of the aerogel. The low intensity of the bands assigned to polyurea can be attributed to the extremely low polyurea content of these materials, which is much lower compared to X-alginate aerogels.^45^ That is because MC has fewer functional (−OH) groups available for the reaction with the triisocyanate compared to alginate, i.e., 1.1–1.5 versus 2 – OH groups, as the degree of substitution (DS) is 1.5–1.9.

**Figure 3:**
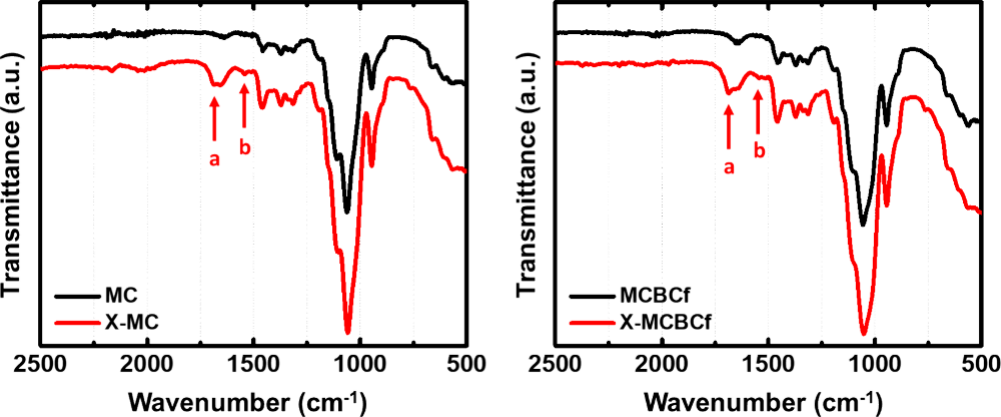
ATR-FTIR spectra of native (MC and MCBCf) and polyurea-cross-linked (X-MC and X-MCBCf) 3D-printed aerogels, as indicated. Arrows (a, b) indicate the characteristic bands discussed in the text (a: 1686 cm^–1^; b:1541 cm^–1^).

XPS and ToF-SIMS were also employed to confirm the formation of polyurea. Figure 4 shows survey and high-resolution XPS spectra for O 1s, C 1s, and N 1s, with an analyzed depth of approximately 5 nm. In X-MC aerogels, the appearance of the N 1s peak clearly indicated the presence of nitrogen-containing species (Figure 4a,d) on the surface of the aerogel. As expected, no N 1s peak could be observed in the spectrum of native MC aerogels, as there are no nitrogen species (Figure 4a,d) in the biopolymer. The binding energies for the O 1s (Figure 4b) and N 1s (Figure 4d) peaks correspond to C = O and imide nitrogen, respectively.^33,60^ No significant difference in the shape of C 1s spectra is present for the two materials (Figure 4c). The above findings confirmed the presence of polyurea on the surface of the skeletal framework.

**Figure 4:**
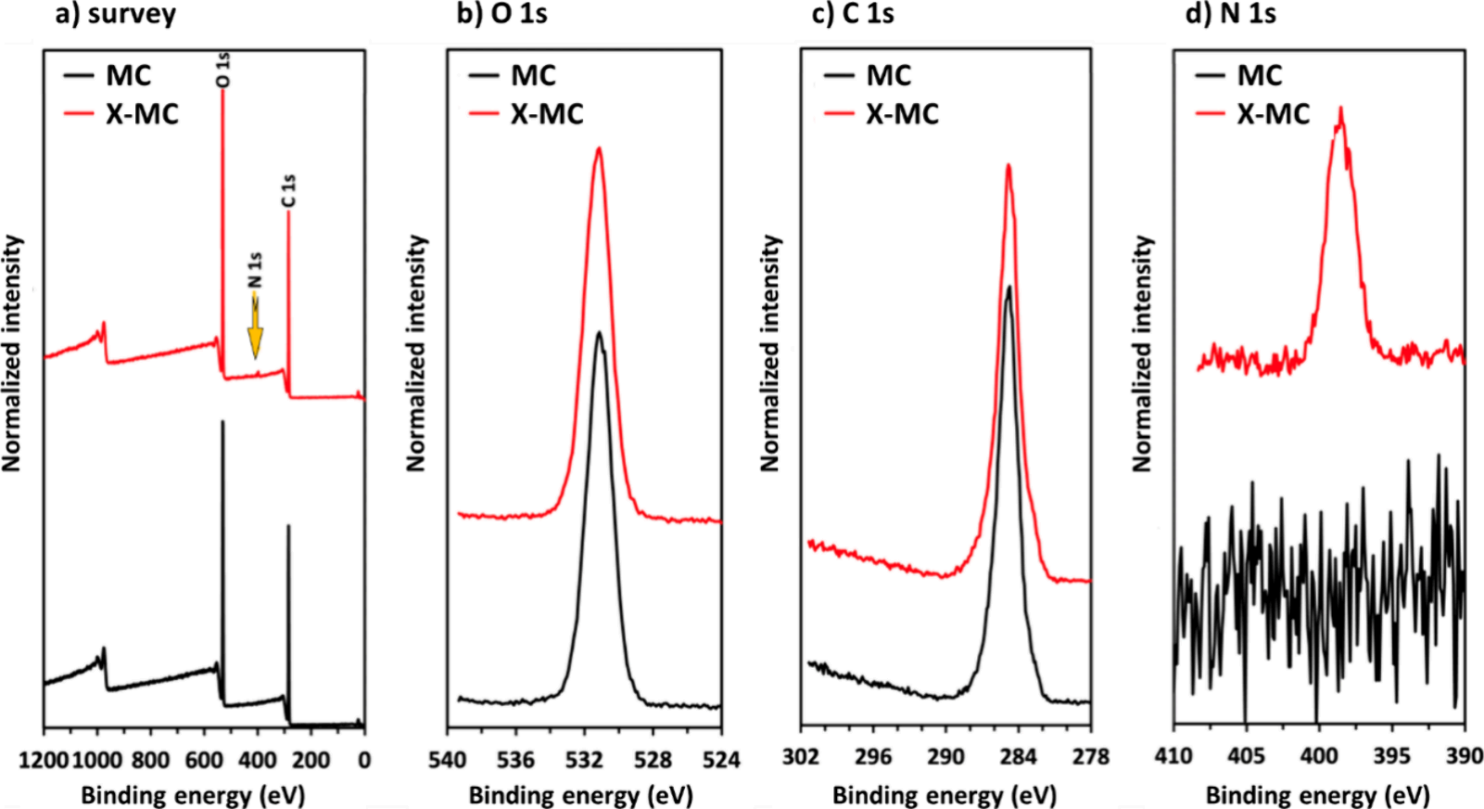
Survey (a) and high-resolution O 1s (b), C 1s (c), and N 1s (d) XPS spectra of native MC and polyurea-cross-linked X-MC 3D-printed cellulose aerogels.

ToF-SIMS analysis was conducted to map the distribution of polyurea and cellulose within the 3D-printed aerogels. In the X-MC aerogel sample, a distinct NH^–^ signal at *m*/*z* 15.01 was detected, indicating the incorporation of nitrogen species during the cross-linking process. On the other hand, this signal was absent in the MC aerogel sample, which agrees with the absence of nitrogen species in MC (Figure 5a). The NH^–^ signal was further utilized to visualize the spatial distribution of polyurea across the aerogel structure (Figure 5c). This mapping revealed that polyurea was dispersed throughout the bulk of the aerogel network. Moreover, regions of higher intensity related to the higher NH^–^ content were randomly distributed throughout the polyurea-functionalized aerogels. In contrast, the C_2_H_3_O^–^ signal at *m*/*z* 59.01 (Figure 5b) corresponded to the presence of cellulose,^61^ which served as the structural matrix of the aerogels. Figure 5c confirms that cellulose in X-MC aerogels is broadly dispersed with only subtle intensity variations throughout the volume of the material. For MC aerogels (Figure 5d), cellulose remains widespread, with noticeable patches with lower signal density, giving the overall distribution a more uneven appearance. The overlapping distributions of polyurea and MC (Figure 5c) confirm the uptake of polyurea by the porous nanostructure.

**Figure 5:**
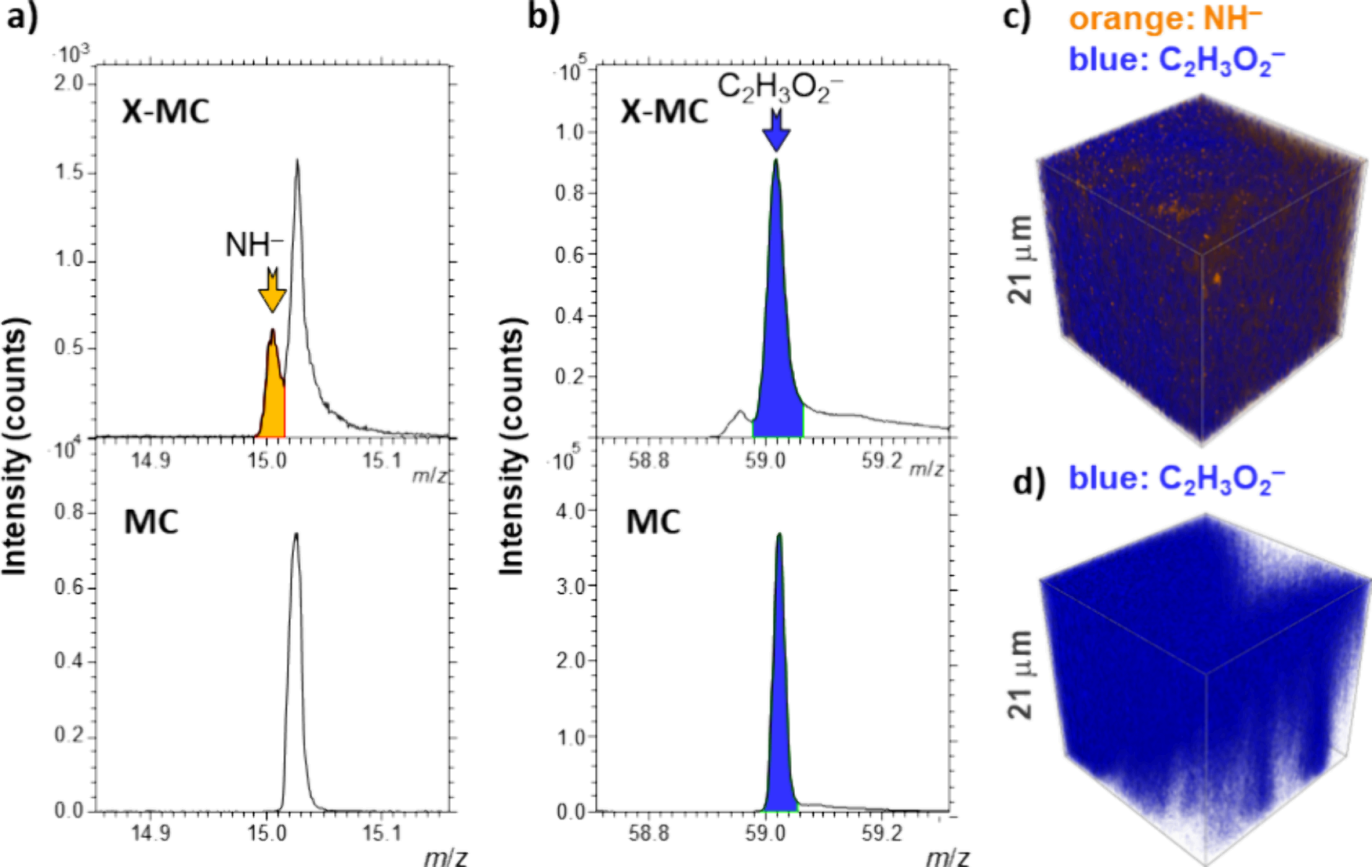
(a) ToF-SIMS spectra highlighting the presence of the NH^–^ signal in the polyurea-cross-linked X-MC aerogels, which is absent in native MC aerogels. (b) ToF-SIMS spectra showing the C_2_H_3_O_2_^–^ signal, indicative of cellulose. (c, d) 3D ToF-SIMS images illustrating the spatial distribution of cellulose (blue, represented by the C_2_H_3_O_2_^–^ signal) and polyurea (orange, represented by the NH^–^ signal) for the (c) X-MC and (d) MC aerogels. The analysis was conducted over an area of 200 by 200 μm (x- and *y*-axis), with a *z*-axis depth of 21 μm, as determined by 3D profilometry following the sputtering procedure.

### 3.3 Selected Material Properties

Selected material properties of native and polyurea-cross-linked aerogels are presented in Table 1 and graphically presented in Figure 6. All 3D-printed aerogels herein reported presented low bulk density (<0.19 g/cm^3^; Figure 6a) and high porosity (>88%; Figure 6b). Interestingly, polyurea-cross-linked aerogels exhibited equal (X-MCBCf) or even higher (X-MC) porosity compared to their native analogues, in contrast to previous observations on X-alginate and X-chitosan aerogels,^45,47^ which can be attributed to the higher shrinkage of native versus cross-linked aerogels during supercritical drying. Indeed, the volume shrinkage (calculated from measuring the dimensions of the aerogels before and after supercritical drying) of X-MC and X-MCBCf aerogels was lower compared to their native counterparts (Figure 6c), which confirms the expected rigidification of the solid framework after cross-linking. The effect is more pronounced for the MC/X-MC pair, since BCf have already enhanced the rigidity of the MCBCf skeleton versus the MC skeleton. The skeletal density of polyurea-cross-linked aerogels is also almost the same (X-MCBCf) or higher (X-MC) compared to that of their native analogues (Figure 6d); the same trend has been reported for glutaraldehyde-cross-linked 3D-printed calcium alginate and calcium alginate/hydroxyapatite aerogels.^25^ This finding shows that cross-linking densifies the skeletal network by bringing the polymeric strands closer together and results in the rigidification of the bulk structure, which, in turn, resists shrinkage, as discussed above. It also agrees with the low polyurea content (as discussed in Section 3.2).

**Table 1:** Selected Material Properties of Native (MC and MCBCf) and Polyurea-Cross-Linked (X-MC and X-MCBCf) 3D-Printed Cellulose Aerogels

**formulation**	**bulk density ρ**_**b**_ **(g cm** ^**–3**^ **)**	**skeletal density ρ**_**s**_ **(g cm** ^**–3**^ **)**	**porosity** ^a^ **Π (% v/v)**	**BET surface areaσ** **(m** ^**2**^ **g** ^**–1**^ **)**	***V*_1.7–300 nm_** ^b^**(***V*_**Total**_**)**^c^**(cm**^**3**^ **g** ^**–1**^ **)**	**average pore diameter** ^d^ **(4** *V*_**Total**_ **/σ)** ^e^ **(nm)**
**MC**	0.18 ± 0.04	1.44 ± 0.02	87	262	0.9 (4.5)	12 (69)
**MCBCf**	0.13 ± 0.01	1.40 ± 0.02	90	265	1.5 (6.4)	20 (96)
**X-MC**	0.13 ± 0.02	1.62 ± 0.05	92	257	1.3 (7.1)	20 (110)
**X-MCBCf**	0.15 ± 0.01	1.44 ± 0.01	90	275	1.4 (6.0)	21 (88)

a Porosity calculated according to the formula: (ρ_s_–ρ_b_)/ρ_s_.

b Cumulative volume of pores between 1.7 and 300 nm from N_2_-sorption data and the Barrett–Joyner–Halenda (BJH) desorption method.

c Total pore volume calculated according to the formula: 1/ρ_b_ - 1/ρ_s_.

d Average pore diameter calculated by the 4*V*/σ method; *V* was set equal to the maximum volume of N_2_ adsorbed along the isotherm as *P*/*P*o → 1.0.

e Average pore diameter calculated by the 4*V*/σ formula; *V* was set equal to *V*_Total_.

**Figure 6:**
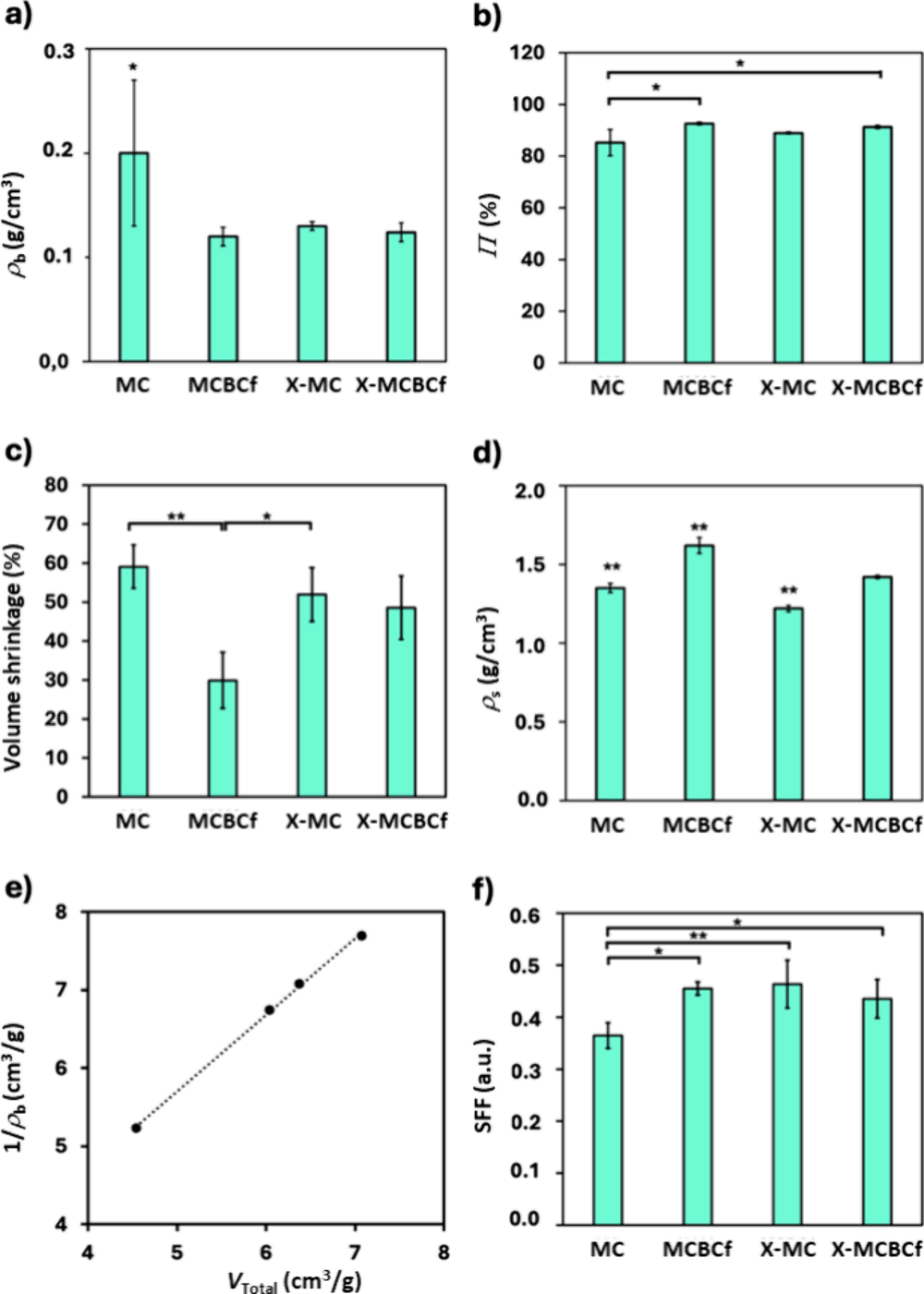
Selected material properties of native (MC and MCBCf) and polyurea-cross-linked (X-MC and X-MCBCf) 3D-printed cellulose aerogels, as indicated. (a) Bulk density (ρ_b_). (b) Porosity (Π). (c) Volume shrinkage (%) during supercritical drying. (d) Skeletal density (ρ_s_). (e) Plot of 1/ρ_b_ versus *V*_Total_ (*R* ^2^ = 0.999). (f) Printing fidelity (SFF). Significant differences among groups were denoted as * or ** (post hoc Tukey HSD multiple comparison test, *p* < 0.05 or *p* < 0.01, respectively).

N_2_ sorption isotherms of all aerogels showed no saturation and narrow hysteresis loops, indicating materials with both mesopores and macropores (Figure 7), in agreement with the *V*_Total_ and *V*_1.7–300 nm_ values from Table 1 (in all cases, *V*_Total_ > *V*_1.7–300 nm_). Microporosity has not been detected from the N_2_ sorption data. In the 1.7–300 nm pore diameter range, the materials were mostly mesoporous, with average pore diameters (by the BJH desorption method) in the range of 12–21 nm. In addition, the plot of 1/ρ_b_ versus *V*_Total_ for all four aerogel formulations of this study is a straight line (Figure 6e), suggesting that there is no closed porosity.^62^

**Figure 7:**
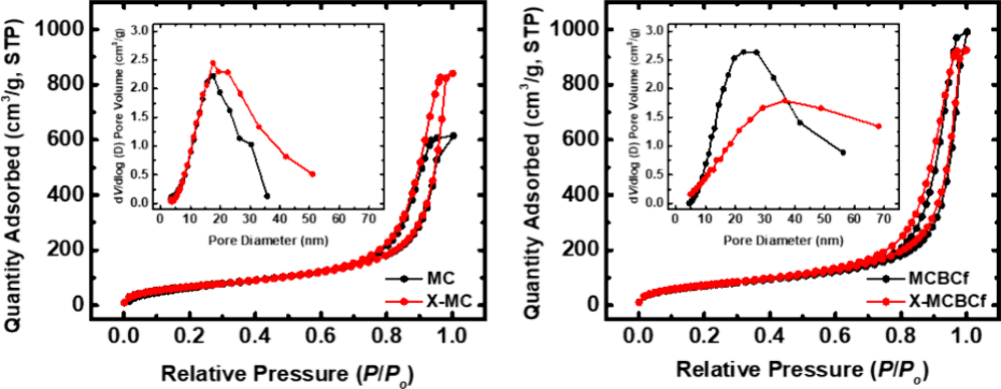
N_2_ sorption isotherms and pore size distributions using the BJH desorption method (insets) of native (MC and MCBCf) and polyurea-cross-linked (X-MC and X-MCBCf) 3D-printed cellulose aerogels, as indicated.

The BET specific surface area of all aerogels was in the range of 257–275 m^2^ g^–1^; no significant differences were observed among native and polyurea-cross-linked materials. This finding indicates that the size of the primary particles of all aerogels is almost the same, although it has been reported for X-alginate aerogels that primary particles become bigger after cross-linking (from 8.3 to 8.8 nm),^58^ and can be attributed to the lower polyurea content of these materials, compared to X-alginate aerogels (as discussed in Section 3.2), which does not coat the biopolymer skeleton, but cross-links the skeletal nanoparticles.

Related to the rigidification of the skeletal network, the printing fidelity (SSF) increased similarly after cross-linking with polyurea (Figure 6f). To the best of our knowledge, this is the first method reported for 3D-printed aerogels able to reinforce the skeleton and improve the printing fidelity without inducing an adverse effect on the physicochemical properties of the porous scaffolds. For example, the reinforcement effect induced by cellulose derivatives incorporated into polylactic acid scaffolds has been reported to induce instability of the ink during the 3D-printing process, decreasing afterward the quality and the resolution.^59^

Polymer-cross-linked aerogels typically incorporate substantial amounts of the cross-linking polymer; for example, X-alginate aerogels uptake 43–97% w/w^45,47^ and X-chitosan aerogels uptake 31–51% w/w^47^ of aliphatic polyurea. However, literature reports have demonstrated that mechanical properties can begin to improve with even lower levels of polymer uptake. For example, in the case of polynorbornene-cross-linked silica aerogels,^38^ mechanical properties started improving, rendering inherently fragile silica aerogels more robust (no longer fragile), after the polymer coated primary particles at approximately 16% w/w, and extremely durable materials were obtained when the polymer filled most of the empty space within secondary particles (26–38% w/w polymer content). Similarly, for polymethylmethacrylate (PMMA)-cross-linked polydicyclopentadiene (PDCPD) aerogels,^43,44^ deformation of PDCPD aerogels was rectified via in situ free radical polymerization of methylmethacrylate (MMA) within the pores of PDCPD gels. Even at a PMMA uptake as low as 13% w/w, the resulting aerogels retained the shape and dimensions of their molds. Since PMMA chain growth is terminated by disproportionation, the enhanced resistance to deformation in the PDCPD/PMMA skeletal framework was not due to molecular-level cross-linking, but rather due to a synergistic interaction governed by the nanotopology of the two components: PMMA filled the space between primary particles, thus halting deformation by preventing penetration of secondary particles into one another.

The X-MC and X-MCBCf aerogels of this work contain a hardly quantifiable amount of cross-linking polymer. Yet, this minimal (just threshold-detectable) incorporation of polyurea was sufficient to reinforce the biopolymer skeleton and significantly enhance mechanical integrity, resulting in exceptional stability in aqueous and physiological environments (as further discussed in the following sections). Importantly, owing to the very small amount of polyurea, the sustainable nature of X-MC and X-MCBCf aerogels remains unaltered, and they are still considered biomaterials. These properties, along with the improved printing fidelity, render these materials promising candidates for biomedical applications, particularly in tissue engineering scaffolding.

### 3.4 Water Uptake and Water Contact Angle Measurements

X-MC and X-MCBCf aerogels showed long-term stability in water; qualitative experiments (based on observation) indicated that the materials can be stable in water for at least 3 months. This observation agrees with the impressive stability of X-alginate aerogels in various aqueous environments, including seawater.^48,49^ On the other hand, MC and MCBCf aerogels exhibited only short-term stability in water. Specifically, MC aerogels were previously drug-loaded and displayed a controlled release for 24 h in PBS medium, thus indicating the maintenance of a certain stability in water-based fluids for this period.^19^

The wetting properties of X-MC and X-MCBCf aerogels were studied via water contact angle and water uptake measurements. Water contact angles were determined to assess the surface hydrophilicity of the materials, as this is correlated with cell adhesion and proliferation and represents an important feature in tissue engineering.^59,63^ The water contact angles for MC, MCBCf, X-MC and X-MCBCf aerogels were found to be equal to 59 ± 5, 84 ± 2, 76 ± 11 and 76 ± 8 degrees, respectively (Figure 8a). The higher hydrophobicity of native MCBCf relative to MC aerogels is attributed to modification of the framework texture imposed by the addition of BCf. Evidently, then, even a very small amount of polyurea on the skeleton of both types of aerogels was sufficient to mask their differences due to surface roughness and bring about convergence of their hydrophilic/hydrophobic properties, as reflected by the common water contact angles (≈76 degrees). For reference, the water contact angle for dense polyurea derived from N3300 is 69.1 degrees,^64^ while the water contact angles for pure polyurea aerogels vary from 67 to 154 degrees, depending on their texture.^33^ Furthermore, by comparison, the water contact angles on other fibrous polyurea-cross-linked polysaccharide-based aerogels, such as X-alginate aerogels with a polyurea content of 50–97% w/w, can reach up to 121 degrees.^45^ In summary, the variation and convergence of the water contact angles for X-MC and X-MCBCf aerogels comprise independent confirmation of the presence of the cross-linking polymer (polyurea) on the biopolymer network.

**Figure 8:**
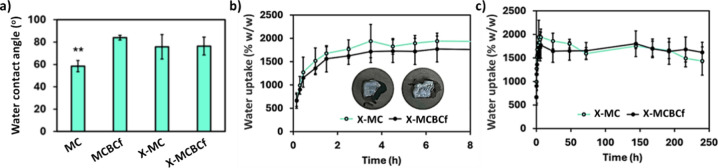
(a) Water contact angles of native (MC and MCBCf) and polyurea-cross-linked (X-MC and X-MCBCf) 3D-printed cellulose aerogels, as indicated. Statistically significant differences among groups are denoted as ** (post hoc Tukey HSD multiple comparison test, *p* < 0.01). (b,c) Water uptake (% w/w) by X-MC and X-MCBCf aerogels, after immersion in water (37 °C, 50 rpm) at different time intervals, as indicated. Frame (b) emphasizes the first 8 h. Inset: Optical photographs of X-MC and X-MCBCf after the water uptake tests, emphasizing the stability of the samples after 10 days.

For the water uptake experiments, preweighed X-MC and X-MCBCf aerogel samples were kept in Eppendorf tubes containing water at 37 °C under constant agitation. At specific time intervals, samples were taken out of the tube and the water uptake was determined according to eq 5. The results are presented in Figure 8b,c. These results also show that the materials can uptake water up to 1500% w/w (X-MC) or 1800% w/w (X-MCBCf) in less than 2 h and then remain stable for 10 days. This very high water uptake falls within the upper range reported for biopolymer aerogels; for example, for food-grade cellulose aerogels the reported range is 400 to 800% w/w,^65^ or for starch aerogels the reported range is 685 to 1714% w/w.^66^ This characteristic is particularly noteworthy and will be further investigated for potential applications in water absorption.

### 3.5 Preliminary Biological Evaluation

The cytocompatibility of X-MC and X-MCBCf aerogels was evaluated by measuring the viability of mouse embryo fibroblasts (NIH/3T3) after contact with sterilized aerogel samples. Cells were seeded with 12,000 cells cm^–2^ in 24-well plates with Dulbecco’s Modified Eagle’s Medium supplemented with bovine calf serum, streptomycin and penicillin. After incubation at 37 °C for 24 or 72 h, the samples were removed, the growth medium was aspirated and a resazurin solution was added to each well. The reduction of blue resazurin (7-hydroxy-3H-phenoxazin-3-one-10-oxide) to fluorescent pink resorufin (7-hydroxy-3H-phenoxazin-3-one) can be induced only by living cells. Therefore, this reaction can be used to assess the cytocompatibility of the aerogels under study.^67^ The viability of the NIH/3T3 cells was calculated from fluorescence measurements of resorufin, according to eq 6, using the resazurin solution as a blank, and cells incubated without contact with aerogels as positive controls. The results are presented graphically in Figure 9a. High cell viability values (>90%) were observed after contact of X-MC and X-MCBCf aerogel samples with the NIH/3T3 cells for 24 or 72h, without statistically significant differences to the controls, indicating that the polyurea-cross-linked aerogels are not cytotoxic.

**Figure 9:**
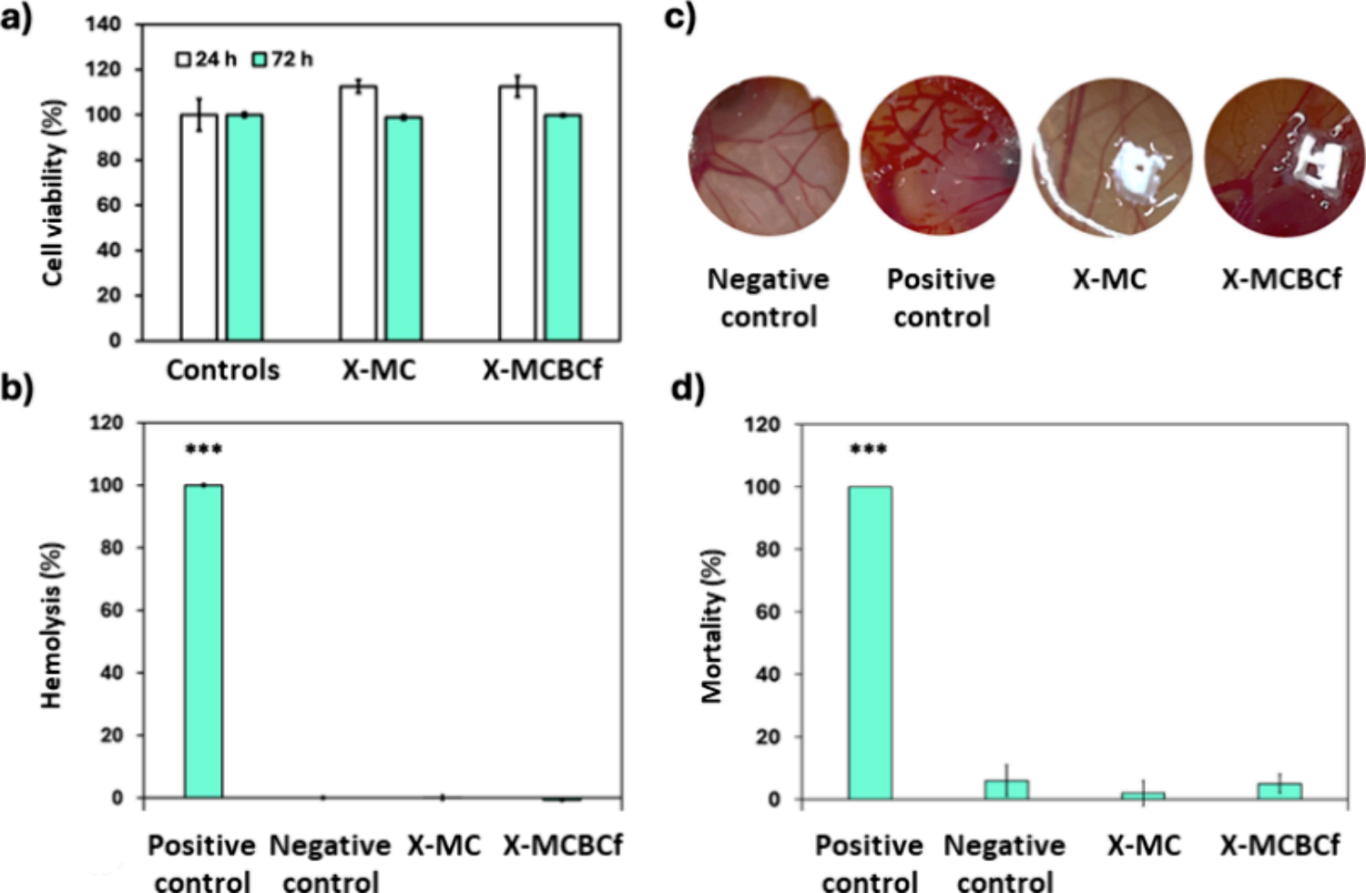
Biological evaluation of polyurea-cross-linked (X-MC and X-MCBCf) 3D-printed cellulose aerogels. (a) Cytocompatibility of NIH/3T3 cells after 24 and 72 h in contact with aerogel samples determined with the resazurin test. Controls: NIH/3T3 cells. (b) Hemolytic activity of X-MC and X-MCBCf aerogels. Negative and positive controls were PBS (pH 7.4) and Triton X-100 (4% *v*/*v*), respectively. (c) HET-CAM test of X-MC and X-MCBCf aerogels. Negative and positive controls were PBS (pH 7.4) and NaOH (0.1 N), respectively. (d) Mortality of *A. salina* after 24 h in contact with X-MC and X-MCBCf aerogels. Negative and positive controls were artificial seawater and DMSO, respectively. Statistically significant differences among groups are represented as *** (post hoc Tukey HSD multiple comparison test; *p* < 0.001).

A key parameter to ensure the successful use of biomaterials as long-term tissue engineering implants and their safe clinical use is their proper interaction with blood cells.^68,69^ For that reason, hemocompatibility tests were carried out using human blood. X-MC and X-MCBCf aerogel samples were kept in fresh human blood diluted with a NaCl solution. After incubation (37 °C, 60 min, 100 rpm) and centrifugation, the absorbance of the hemoglobin was measured in the supernatant, and hemolysis was calculated according to eq 7 (phosphate buffer saline and Triton X-100 were also added as negative and positive controls, respectively). As shown in Figure 9b, no hemolytic activity was detected after contact of X-MC and X-MCBCf aerogel samples with human blood.

In addition, the HET-CAM test was employed to evaluate the irritative potential of X-MC and X-MCBCf aerogels after implantation.^19^ Fertilized hens’ eggs were incubated for 9 days before X-MC and X-MCBCf aerogel samples were in contact with CAM. CAM vessels were observed for the standard test time (5 min after contact with the aerogel samples) to visually check potential hemorrhage, vascular lysis, or clotting (Figure 9c). From the images of Figure 9c, where a comparison with the positive (NaOH) and negative (PBS) controls can also be made, it is obvious that no hemorrhage, vascular lysis, or clotting could be observed due to X-MC and X-MCBCf aerogels, further confirming the hemocompatibility of these materials.

The brine shrimp *A. salina* was selected as a preliminary *in vivo* model to evaluate the safety of X-MC and X-MCBCf aerogels. *A. salina* has been described as a suitable method to evaluate the acute toxicity of different biomaterials^70,71^ and also as a screening method to evaluate the safety of nanoparticles (especially their environmental impact).^72^ *A. salina* eggs were left in artificial seawater to hatch for 48 h under aeration and illumination at 25–30 °C. Then, artificial seawater containing 10–15 nauplii was poured into each well of a 24-well plate and X-MC and X-MCBCf aerogel samples, artificial seawater (negative control) or DMSO (positive control) were added and incubated for 24 h. The dead nauplii were counted. Afterward, DMSO was pipetted to kill all living nauplii. The mortality was calculated according to eq 8. As shown in Figure 9d, statistically significant differences were observed between the mortality of the *A. salina* of the positive control (DMSO) and the experiments with X-MC and X-MCBCf aerogel samples. Similar results from previous studies, evaluating different cellulose aerogel formulations, were related to the preliminary safety of the biomaterial.^19,24^

The results of the biological evaluation of X-MC and X-MCBCf aerogels agree with previous *in vitro*, *ex vivo* and *in vivo* studies on X-silica and X-alginate aerogels. More specifically, X-silica aerogels did not affect the cell viability when cocultured with bone marrow microvascular endothelial cells and human umbilical vein endothelial cells for 5 days, which suggested that these materials are good candidates for blood implantable devices.^73^ Their *in vivo* evaluation *via* long-term (up to 20 months) subcutaneous and intramuscular implantation in Sprague–Dawley rats did not induce significant inflammatory responses and were well-tolerated by the surrounding tissues or distant organs, thereby confirming the absence of toxicity and the high tolerance of the aerogels.^57^ Linked to the above, their potential utilization as neuronal scaffolds has been showcased by the enhanced neurite extension of PC-12 cells cultured on X-silica aerogel substrates rather than on traditional tissue-culture substrates,^74^ as well as by growth and proliferation of various cell types, including proximal tubule-like opossum kidney cells over extended periods without cytotoxic effects.^4^ X-alginate aerogels are also good candidates for implants, as they have shown tunable biodegradability under physiological conditions, both *in vitro* and *ex vivo*,^52^ with the degradation rate depending on the polyurea content of the aerogels, which can be easily adjusted just by modifying the synthetic parameters. Last, but not least, X-silica and X-alginate aerogel implants can be monitored with ultrasound imaging,^53,75^ thus providing a facile and straightforward way to monitor the stability and/or biodegradation of the implants.

## 4 Conclusions

The most important findings of this work are

a) Biopolymer aerogels in complex form factors have been prepared by 3D-printing and stabilized at the nanoscopic level with the X-aerogel technology (implemented by cross-linking the skeletal framework with polyurea). Both technologies are quite new in the preparation of biopolymer-based aerogels, with only a few prior examples in the literature, and have never been used together before. More specifically, preformed 3D-printed cellulose (MC and MCBCf) gels were cross-linked with an aliphatic polyurea (from Desmodur N3300).
b) Polyurea-cross-linked (X-MC and X-MCBCf) aerogels are highly porous materials (porosity >90% v/v), with bimodal hierarchical porosity comprising interconnected macropores and mesopores, and no closed porosity. Their BET surface areas are in the range of 257–275 m^2^ g^–1^. Their material properties are similar to those of their native counterparts. However, by comparison, their skeletal network has been rigidized after cross-linking, leading to lower volume shrinkage, lower bulk density, and higher skeletal density.
c) The uptake of polyurea by the biopolymer skeleton was evidenced by ATR-FTIR, XPS and ToF-SIMS. The cross-linking process did not affect the morphology of the aerogels, including bimodal hierarchical porosity, as shown with SEM.
d) Another important finding of this work is the efficient homogenization of BCf in the 3D-printing ink, which resulted in gels and aerogels with a homogeneous distribution of BCf (as shown with TEM and confocal microscopy of SPIONs-doped materials) and a more rigid structure.
e) Related to the rigidification of the skeletal network, the printing fidelity (in terms of SSF) was increased after cross-linking with polyurea. To the best of our knowledge, this is the first method reported for 3D-printed aerogels able to reinforce the skeleton and improve the printing fidelity without inducing an adverse effect on the physicochemical properties of the porous scaffolds.
f) X-MC and X-MCBCf aerogels show long-term stability in water. Although the water contact angles for X-MC and X-MCBCf aerogels are <90 degrees, polyurea-cross-linking makes these structures resistant to collapse after contact with water. These aerogels can uptake water up to 1500% w/w (X-MC) or 1800% w/w (X-MCBCf) in less than 2 h and then remain stable for 10 days. Overall, these comprise an independent confirmation of the presence of the cross-linking polymer (polyurea) on the biopolymer network, contributing to modifications in surface properties.
g) A preliminary biological evaluation (cytocompatibility, using NIH/3T3 cells, hemocompatibility, HET-CAM, and preliminary safety, using *A. salina*, tests) was performed to ensure the biocompatibility of X-MC and X-MCBCf aerogels, with good results in terms of cell viability, blood compatibility and preliminary safety for living organisms.
h) This work demonstrates, for the first time, that even a minimal (barely detectable) amount of polyurea is sufficient to enhance the dimensional integrity of the biopolymer framework and render the resulting aerogels resilient against disintegration not only in pure water, but also in physiological environments. This finding is extremely important as all previously reported X-aerogels in the literature carry substantial amounts of cross-linking polymer, in the range of 13–97% w/w. Collectively, the findings of this study position X-MC and XMCBCf aerogels as sustainable and promising candidates for tissue engineering scaffolds and support further exploration of their potential in biomedical applications.

## d

Unitat de Biofísica i Bioenginyeria, Facultat de Medicina i Ciències de la Salut,Universitat de Barcelona, 08036, Barcelona, Spain
